# Second-trimester fetal thymic-thoracic ratio in pregnancies treated with levothyroxine for elevated first-trimester TSH: a retrospective case-control study

**DOI:** 10.61622/rbgo/2026rbgo80

**Published:** 2026-10-30

**Authors:** Fatma Nazlı Demir, Zeycan Kübra Cevval, Umutcan Kayıkçı

**Affiliations:** 1 Ministry of Health Kastamonu Research and Training Hospital Department of Obstetrics and Gynaecology Kastamonu Turkey Ministry of Health Kastamonu Research and Training Hospital, Department of Obstetrics and Gynaecology, Kastamonu, Turkey.; 2 Kastamonu University Faculty of Medicine Department of Radiology Kastamonu Turkey Kastamonu University Faculty of Medicine, Department of Radiology, Kastamonu, Turkey.; 3 Ministry of Health Kastamonu Research and Training Hospital Department of Obstetrics and Gynaecology Division of Maternal and Fetal Medicine Kastamonu Turkey Ministry of Health Kastamonu Research and Training Hospital, Department of Obstetrics and Gynaecology, Division of Maternal and Fetal Medicine, Kastamonu, Turkey.

**Keywords:** Thymus gland, Thymic-thoracic ratio, Thyroid-stimulating hormone, Thyroxine, Thyrotropin, Pregnancy, Pregnant people, Pregnancy trimester, second, Pregnancy trimester, first, Gestational age, Ultrasonography

## Abstract

**Objective:**

Maternal thyroid dysfunction during pregnancy has been associated with adverse fetal and obstetric outcomes; however, the relationship between early pregnancy TSH elevation, levothyroxine treatment, and fetal thymic development remains unclear. This study aimed to compare second-trimester fetal thymic-thoracic ratio (TTR) values between pregnancies receiving levothyroxine after elevated first-trimester thyroid-stimulating hormone (TSH) levels and healthy controls.

**Methods:**

This retrospective case-control study included 109 pregnant women who underwent second-trimester obstetric ultrasonography between February 2023 and December 2024. Pregnancies treated with levothyroxine after first-trimester TSH levels ≥2.5 mIU/L (n=43) were compared with healthy controls without thyroid disease or pregnancy-related complications (n=66). Fetal thymic-thoracic ratio measurements were obtained from stored ultrasound images using the method described by Chaoui et al. Continuous variables were compared using the Mann–Whitney U test.

**Results:**

No statistically significant difference was observed in fetal thymic-thoracic ratio values between the levothyroxine-treated group and the healthy control group (median TTR: 0.420 [0.345–0.445] vs. 0.395 [0.323–0.450], respectively; p=0.483). Similarly, no significant differences were found between the groups regarding maternal age, gestational age at examination, thymic diameter, thoracic diameter, BMI, or smoking status (all p>0.05).

**Conclusion:**

Second-trimester fetal TTR values did not differ significantly between healthy controls and pregnancies treated with levothyroxine after elevated first-trimester TSH. These preliminary findings suggest that early-treated maternal TSH elevation may not be associated with measurable alterations in fetal thymic size during the second trimester. However, the retrospective design, incomplete characterization of thyroid status (including free T4, thyroid antibodies, levothyroxine dose, and confirmation of euthyroidism), and limited statistical power preclude definitive conclusions regarding the effect of treatment. Further prospective, adequately powered longitudinal studies with complete thyroid function data are needed to clarify this relationship.

## Introduction

Thyroid disorders are among the most common endocrine conditions encountered in women of reproductive age and may become clinically relevant during pregnancy because of the increased maternal demand for thyroid hormones.^(1)^ Maternal thyroid hormones are essential for early fetal growth, organogenesis, and neurodevelopment. During the first trimester, before the fetal thyroid gland becomes functionally mature, the fetus is largely dependent on transplacental maternal thyroxine transfer.^(2)^ Therefore, even mild disturbances in maternal thyroid function during early gestation may have potential implications for fetal development.

Adequate maternal thyroid hormone availability is essential for an uncomplicated pregnancy and optimal fetal development. Overt thyroid disease has been associated with adverse obstetric and fetal outcomes, including miscarriage, hypertensive disorders of pregnancy, preterm birth, fetal growth abnormalities, and neurodevelopmental impairment.^(3)^ However, the clinical significance and management of mild TSH elevation or subclinical thyroid dysfunction during pregnancy remain more controversial. Several guidelines and clinical protocols have historically used 2.5 mIU/L as an upper reference threshold for first-trimester TSH, while treatment thresholds vary according to thyroid autoantibody status, degree of TSH elevation, local reference ranges, and clinical context.^(4)^ Therefore, studies evaluating fetal effects of maternal TSH elevation should consider not only the initial TSH value but also treatment status and the timing of fetal assessment. Recent studies have also suggested that mild maternal thyroid dysfunction during early pregnancy may be associated with long-term offspring neurodevelopmental outcomes, further supporting the importance of early identification and appropriate clinical management.^(5)^

The thymus is a primary lymphoid organ responsible for T-lymphocyte maturation, central immune tolerance, and the development of adaptive immune competence. Immature T cells originating from the bone marrow undergo differentiation and selection within the thymic microenvironment, allowing the generation of functional T cells capable of recognizing foreign antigens while maintaining tolerance to self-antigens.^(6)^ Because thymic development may be influenced by intrauterine stress, endocrine signals, inflammation, and fetal growth conditions, prenatal thymus assessment has emerged as a potential sonographic marker of fetal immune and stress-related adaptation.

In recent years, prenatal evaluation of the fetal thymus has become an area of growing interest in obstetric ultrasonography. On ultrasound, the fetal thymus can be visualized as a homogeneous hypoechoic structure located in the anterior mediastinum on the three-vessel and trachea view. The thymic-thoracic ratio (TTR), obtained by dividing the anteroposterior thymic diameter by the corresponding anteroposterior mediastinal thoracic diameter, provides a simple and reproducible approach for assessing fetal thymic size while partially accounting for overall thoracic dimensions. Reduced fetal thymus size has been reported in pregnancies complicated by chorioamnionitis, preeclampsia, gestational diabetes mellitus, intrauterine growth restriction, and other inflammatory or stress-related conditions.^(7–9)^

Although previous studies have evaluated fetal thymic size in pregnancies complicated by maternal thyroid disorders, the findings remain inconsistent, possibly because of differences in thyroid autoimmunity, treatment status, gestational age at assessment, and disease activity at the time of fetal thymus measurement. Importantly, most available studies, including the present one, characterize maternal thyroid status using first-trimester TSH values and treatment status alone, without complete information on free T4 concentrations, thyroid autoantibody status, levothyroxine dose, or confirmation of biochemical euthyroidism at the time of fetal assessment; findings in this field should therefore generally be interpreted as reflecting a first-trimester TSH elevation that prompted treatment, rather than a fully characterized thyroid disease state.

Therefore, this study aimed to compare second-trimester fetal thymic-thoracic ratio values between healthy controls and pregnancies receiving levothyroxine treatment after elevated first-trimester TSH levels.

## Methods

This retrospective case-control study was conducted using the medical records and stored ultrasound images of pregnant women who attended the Department of Obstetrics and Gynecology at Ministry of Health Kastamonu Research and Training Hospital between February 2023 and December 2024. Maternal demographic characteristics, obstetric history, first-trimester thyroid-stimulating hormone (TSH) levels, and levothyroxine treatment status were obtained from hospital records.

Pregnancies with first-trimester TSH levels ≥2.5 mIU/L who received levothyroxine treatment after endocrinology/internal medicine consultation were assigned to the levothyroxine-treated elevated TSH group. The 2.5 mIU/L threshold was chosen because it corresponds to the upper first-trimester TSH reference limit historically recommended by earlier American Thyroid Association and endocrine society guidance and was the threshold applied for endocrinology referral and treatment decisions in clinical practice at our institution during the study period. Healthy pregnant women without thyroid disease, thyroid medication use, or pregnancy-related complications served as controls. A total of 109 pregnancies were included: 43 in the levothyroxine-treated elevated TSH group and 66 in the healthy control group. Data on free thyroxine (FT4) concentrations, thyroid autoantibody status, the specific indication for treatment, levothyroxine dose, and confirmation of biochemical euthyroidism before the ultrasound examination were not uniformly available in the hospital records and could therefore not be incorporated into the group definition; consequently, the levothyroxine-treated group is defined by a first-trimester TSH value above a fixed threshold followed by treatment initiation, rather than by a fully biochemically characterized thyroid disease state, and this should be borne in mind when interpreting all subsequent results.

Cases with missing clinical data, inadequate ultrasound image quality, fetal structural or chromosomal anomalies, preeclampsia, pregestational diabetes mellitus, abnormal amniotic fluid volume, premature rupture of membranes, corticosteroid use, or other significant maternal systemic diseases were excluded. This retrospective study was approved by the Ethics Committee of Kastamonu University Faculty of Medicine (approval no: 2024-KAEK-7).

### Ultrasound examination and fetal thymic-thoracic ratio measurement

All examinations were performed using a Samsung V8 ultrasound system (Samsung Medison, Seoul, Republic of Korea) by two experienced physicians a perinatologist and a radiologist with 10 years of experience in obstetric ultrasonography following the institutional protocol. During retrospective image evaluation, fetal thymic measurements were performed by one examiner using stored images and subsequently reviewed by a second experienced examiner to ensure measurement consistency. Stored ultrasound images obtained during routine anomaly screening were retrospectively reviewed. Fetal thymic-thoracic ratio (TTR) measurements were performed using the three-vessel and trachea view according to the method described by Chaoui et al.,^(10)^ as illustrated schematically and demonstrated on a representative ultrasound image in figure 1. The thymic anteroposterior diameter was measured between the posterior sternum and great vessels, while the thoracic diameter was measured between the sternum and anterior vertebral body along the same axis. TTR was calculated by dividing thymic diameter by thoracic diameter. Two measurements were obtained for each fetus, and the mean value was used for analysis.

**Figure 1:**
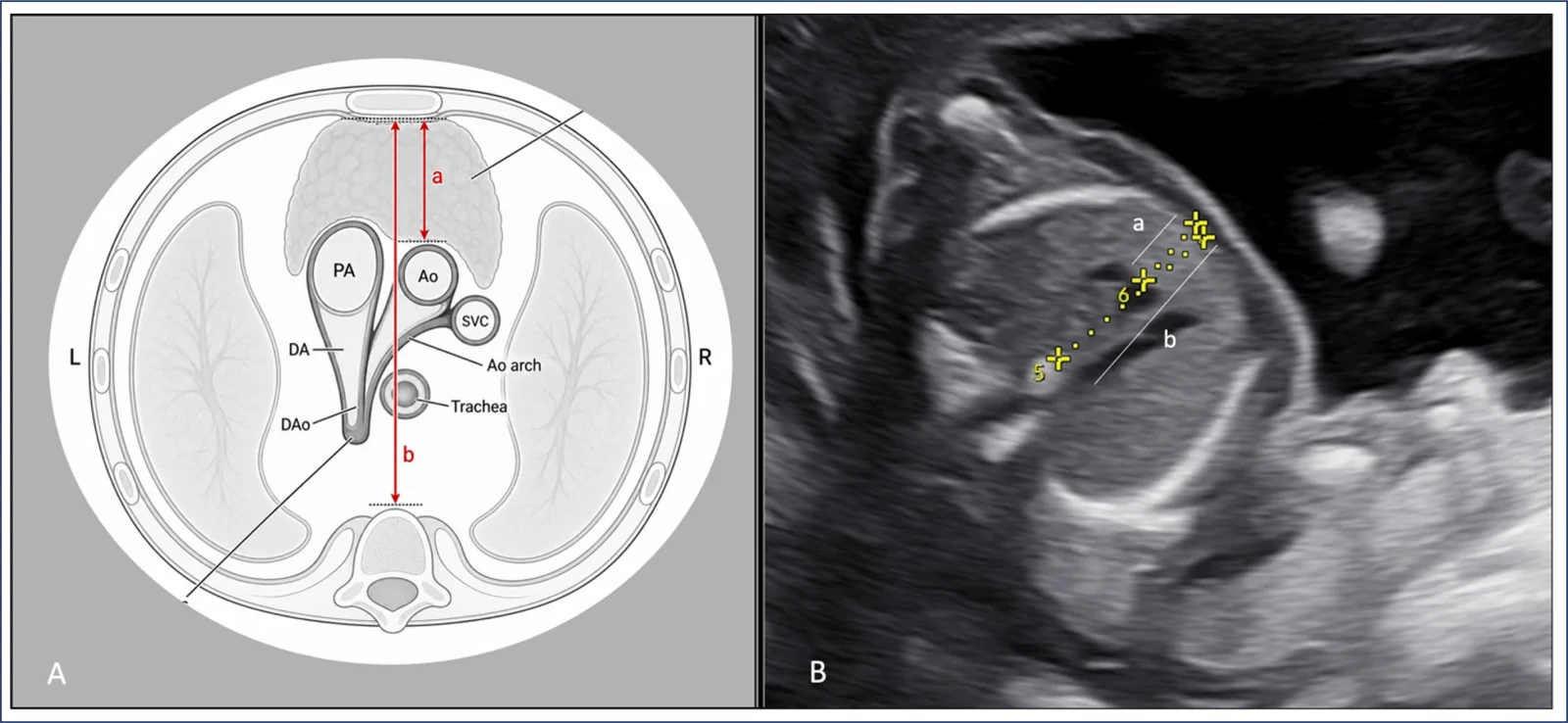
Fetal thymic-thoracic ratio (TTR = a/b) measurement in the three-vessel and trachea view

(A) Schematic illustration. (B) Representative ultrasound image. Line a indicates the distance between the sternum and the great vessels, and line b indicates the distance between the sternum and the anterior border of the vertebral body

### Statistical analysis

Statistical analyses were performed using Python software with the SciPy library. Continuous variables were assessed for normality using the Shapiro–Wilk test. Because TTR values were not normally distributed, continuous variables were compared using the Mann–Whitney U test. Categorical variables were compared using Fisher's exact test when appropriate. Continuous variables are presented as median (25th–75th percentile), and categorical variables as number and percentage. A p-value <0.05 was considered statistically significant. Given the relatively small size of the levothyroxine-treated elevated TSH group (n=43) relative to the number of potentially relevant covariates, multivariable regression modeling was not performed, as it would have carried a high risk of overfitting and unstable effect estimates; group comparisons were therefore restricted to univariate non-parametric tests, and adjustment for potential confounders (e.g., gravidity) is identified as a priority for future, adequately powered studies.

## Results

A total of 109 pregnant women were included in the study. Participants were divided into two groups: the levothyroxine-treated elevated TSH group, consisting of pregnancies with first-trimester TSH levels ≥2.5 mIU/L who received levothyroxine treatment (n=43), and the healthy control group, consisting of pregnant women without known thyroid disease, thyroid medication use, or pregnancy-related complications (n=66).

The primary outcome was the comparison of fetal thymic-thoracic ratio (TTR) values between the levothyroxine-treated elevated TSH group and the healthy control group. Secondary comparisons included maternal demographic characteristics, obstetric history, smoking status, gestational age at examination, BMI, thymic diameter, and thoracic diameter. No statistically significant difference was observed in fetal TTR between the healthy control group and the levothyroxine-treated elevated TSH group. Median TTR was 0.395 (0.323–0.450) in healthy controls and 0.420 (0.345–0.445) in the levothyroxine-treated elevated TSH group (Mann–Whitney U test, p = 0.483). Maternal age, gestational age at examination, anteroposterior thymic diameter, anteroposterior thoracic diameter, BMI, and smoking status were comparable between the healthy control group and the levothyroxine-treated elevated TSH group (all p > 0.05). As expected, first-trimester TSH levels were significantly higher in the levothyroxine-treated elevated TSH group than in healthy controls (p < 0.001). Among obstetric history variables, gravidity differed significantly between the groups (Mann–Whitney U test, p = 0.020), with lower gravidity observed in the levothyroxine-treated elevated TSH group. Parity showed a borderline difference in the same direction (p = 0.063), with a higher proportion of nulliparous women in the levothyroxine-treated elevated TSH group than in healthy controls (65.1% vs. 48.5%) (Table 1 and 2).

**Table 1:** Overall demographic, clinical, and ultrasonographic characteristics of the study population

Characteristics		n(%) or Median (25th–75th Percentiles)
Age (years)		30 (27–33)
Anteroposterior thymic diameter (mm)		8.20 (7.00–9.20)
Anteroposterior thoracic diameter (mm)		20.60 (19.00–22.00)
Thymic-thoracic ratio (TTR)		0.410 (0.330–0.450)
Gestational age at examination		21 (20–21)
Gravidity	1	50(45.9)
	2	33(30.3)
	≥3	26(23.9)
Parity	0	60(55.0)
	1	32(29.4)
	2	15(13.8)
	≥3	2(1.8)
Smoking	No	105(96.3)
	Yes	4(3.7)
TSH (mIU/L)		2.01 (1.48–3.63)
BMI (kg/m²)		25.30 (23.40–29.00)
Groups	Healthy control group	66(60.6)
	Levothyroxine-treated elevated TSH group	43(39.4)

Values are presented as median (25th–75th percentile) for continuous variables and n(%) for categorical variables. TTR: thymic-thoracic ratio; TSH: thyroid-stimulating hormone; BMI: body mass index

**Table 2:** Comparison of demographic, clinical, and ultrasonographic characteristics between the levothyroxine-treated elevated TSH group and healthy controls

Characteristics		Groups		Test	p-value
		Healthy control group (n = 66) Median (Q1–Q3) or n(%)	Levothyroxine-treated elevated TSH group Median (Q1–Q3) or n(%)		
Age (years)		30.0 (27.0–33.8)	29.0 (26.5–33.0)	MWU	0.435
Anteroposterior thymic diameter (mm)		8.00 (6.62–9.17)	8.30 (7.33–9.65)	MWU	0.247
Anteroposterior thoracic diameter (mm)		20.50 (19.00–22.00)	21.00 (19.40–22.50)	MWU	0.427
Thymic-thoracic ratio (TTR)		0.395 (0.323–0.450)	0.420 (0.345–0.445)	MWU	0.483
Gestational age at examination		21 (20–21)	21 (20–22)	MWU	0.900
TSH (mIU/L)		1.54 (1.25–1.89)	4.04 (3.38–4.56)	MWU	<0.001
BMI (kg/m²)		25.05 (23.18–28.98)	26.10 (23.85–29.30)	MWU	0.464
Gravidity (no)^a^	1	26(39.4)	24(55.8)	MWU	0.020
	2	19(28.8)	14(32.6)		
	≥3	21(31.8)	5(11.6)		
Parity (no)^a^	0	32(48.5)	28(65.1)	MWU	0.063
	1	21(31.8)	11(25.6)		
	2	11(16.7)	4(9.3)		
	≥3	2(3.0)	0(0.0)		
Smoking	No	64(97.0)	41(95.3)	Fisher	0.646
	Yes	2(3.0)	2(4.7)		

Values are presented as median (25th–75th percentile) or n(%), as appropriate. Continuous variables were compared using the Mann–Whitney U test. Smoking status was compared using Fisher's exact test.

a Gravidity and parity were analyzed as ordinal variables using the Mann–Whitney U test. p < 0.05 was considered statistically significant. TTR: thymic-thoracic ratio; TSH: thyroid-stimulating hormone; BMI: body mass index

## Discussion

In this retrospective case-control study, second-trimester fetal thymic-thoracic ratio (TTR) values were compared between healthy controls and pregnancies treated with levothyroxine after elevated first-trimester TSH levels. The main finding was that fetal TTR did not differ significantly between the groups. This suggests that pregnancies receiving levothyroxine after early TSH elevation did not show measurable alterations in fetal thymic size during the second trimester compared with healthy controls.

An important feature of our study is that the elevated TSH group consisted of pregnancies in which levothyroxine treatment had been initiated after first-trimester TSH elevation. Therefore, our findings should be interpreted in the context of treated early TSH elevation rather than untreated or active maternal hypothyroidism. Although it is plausible that thyroid management during pregnancy may influence fetal thymic development, our retrospective data do not allow confirmation of biochemical euthyroidism at the time of fetal thymus measurement. Accordingly, the absence of a significant TTR difference should be interpreted cautiously and should not be considered direct evidence of a treatment effect.

The effect of maternal thyroid dysfunction on fetal thymus development has not yet been clearly established. Albayrak et al. reported significantly lower TTR values in pregnancies with subclinical hypothyroidism and demonstrated a negative correlation between maternal TSH and fetal TTR values.^(11)^ In contrast, we did not observe a significant reduction in fetal TTR among pregnancies receiving levothyroxine after elevated first-trimester TSH. One possible explanation for this discrepancy is the difference in disease activity and treatment status at the time of fetal thymus assessment. Active or untreated TSH elevation may have different fetal implications than early detected TSH elevation followed by levothyroxine treatment. However, because contemporaneous thyroid function data were not available for all participants in our study, this explanation remains hypothetical.

Fetal thymic size has been studied as a sonographic marker of intrauterine stress, inflammation, and fetal adaptive responses. Reduced fetal thymus size has been reported in pregnancies complicated by chorioamnionitis, preeclampsia, fetal growth restriction, and diabetic disease, suggesting that thymic involution may reflect activation of fetal stress and inflammatory pathways.^(7,12,13)^ Proposed mechanisms include activation of the fetal hypothalamic–pituitary–adrenal axis, increased exposure to glucocorticoids, and inflammatory cytokine-mediated effects on thymic tissue. In this context, the absence of reduced TTR in our levothyroxine-treated elevated TSH group may indicate that early-treated TSH elevation does not produce a fetal stress response comparable to inflammatory or placental disorders. However, this interpretation remains speculative because inflammatory markers and fetal immune outcomes were not evaluated. The role of thyroid autoimmunity should also be considered when interpreting the relationship between maternal thyroid status and fetal thymic development. Kasap et al. reported higher fetal TTR values in pregnancies complicated by Hashimoto's thyroiditis compared with healthy controls.^(14)^ This finding suggests that thyroid autoimmunity may influence fetal thymic size through mechanisms that are not identical to those observed in classical inflammatory or placental stress conditions. Differences in gestational age at assessment, thyroid antibody status, treatment exposure, and residual thyroid dysfunction may partly explain the heterogeneity among published findings. In our study, thyroid autoantibody data were not available for all participants; therefore, we could not evaluate whether autoimmune thyroiditis modified the association between maternal TSH elevation and fetal TTR.

Our findings are broadly consistent with the study by Yang et al., which reported no significant impairment in fetal thymic development among pregnancies with maternal hypothyroidism receiving levothyroxine treatment.^(15)^ In that study, fetal thymic parameters increased with advancing gestational age, while no significant differences were observed between levothyroxine-treated pregnancies and healthy controls. Together with our findings, these data may suggest that fetal thymic development is not substantially altered in pregnancies managed with levothyroxine. Nevertheless, this interpretation should be made cautiously, as differences in study design, thyroid function monitoring, gestational age at assessment, and treatment protocols may affect comparability between studies.

The clinical interpretation of an absent between-group difference in fetal TTR also warrants discussion. TTR is a proxy measure of thymic size rather than a direct assessment of fetal immune function, and a preserved TTR does not exclude subtler alterations in T-cell output, thymic microarchitecture, or postnatal immune competence that ultrasound cannot capture. From a clinical standpoint, our findings may be reassuring in that early identification and levothyroxine treatment of first-trimester TSH elevation did not appear to be associated with a measurable reduction in fetal thymic size, which would be consistent with a benefit of timely treatment; however, because the study was underpowered to exclude small effects and lacked biochemical confirmation of euthyroidism, this reassurance should be regarded as provisional rather than conclusive. Establishing whether a preserved second-trimester TTR translates into normal postnatal immune outcomes will require studies that link prenatal thymic measurements to neonatal and infant immune parameters.

Overall, the available literature demonstrates considerable heterogeneity regarding the direction and magnitude of the association between maternal thyroid dysfunction and fetal thymic development. This heterogeneity may be explained by differences in study design, gestational age at thymus assessment, thyroid autoantibody status, treatment exposure, disease activity at the time of ultrasound examination, and the use of different thymic measurement techniques. Therefore, fetal thymus findings in maternal thyroid disorders should be interpreted according to the specific clinical phenotype rather than thyroid dysfunction as a single homogeneous entity.

The main strengths of our study include the use of a standardized and previously described TTR measurement method, assessment of fetal thymic size within a relatively narrow second-trimester gestational age range, and comparability between groups with respect to maternal age, gestational age at examination, BMI, and smoking status. In addition, the exclusion of pregnancies complicated by major obstetric, systemic, structural, or chromosomal abnormalities allowed a more focused evaluation of the relationship between levothyroxine-treated early TSH elevation and fetal TTR.

In conclusion, this study did not demonstrate a significant relationship between maternal TSH levels and fetal thymus size, suggesting that treated thyroid dysfunction may have a limited effect on fetal thymus development. Considering the conflicting findings in the literature, it is clear that this relationship should be evaluated together with factors such as autoimmunity, inflammation, and treatment status. Future studies with larger sample sizes, longitudinal designs, and multivariable analyses will help to further clarify the impact of maternal thyroid function on fetal immune development.

This study has several limitations. First, its retrospective design limited the availability of detailed clinical, biochemical, and treatment-related data. In particular, thyroid autoantibody levels, including anti-thyroid peroxidase and anti-thyroglobulin antibodies, levothyroxine treatment doses, treatment duration, treatment adherence, free thyroxine levels, and maternal TSH values measured at the time of fetal thymus assessment were not available for all participants. Therefore, we could not confirm whether all women in the levothyroxine-treated elevated TSH group had achieved biochemical euthyroidism at the time of ultrasound evaluation.

Second, because only stored ultrasound images with adequate visualization of the fetal thymus and surrounding anatomical landmarks were included, selection bias related to image quality cannot be excluded. In addition, fetal thymus measurements were performed retrospectively on archived images rather than prospectively acquired standardized thymus images. Although all measurements were obtained using the same predefined anatomical landmarks, prospective image acquisition could provide more robust measurement standardization.

Third, the sample size was relatively limited, particularly in the levothyroxine-treated elevated TSH group. Therefore, the study may not have been sufficiently powered to detect small differences in fetal TTR between groups. The absence of a statistically significant difference should therefore be interpreted as lack of evidence for a measurable TTR alteration in this sample, rather than definitive evidence of no effect. A post hoc power calculation indicated that, with 43 and 66 participants in the levothyroxine-treated and healthy control groups, respectively, our study had approximately 72% power to detect a medium effect size (Cohen's d=0.5) at α=0.05, and only a between-group effect of d≈0.55 or larger could be reliably detected with 80% power; smaller true differences in fetal TTR may therefore have gone undetected because of limited sample size.

Fourth, this was a single-center study, which may limit the generalizability of the findings. Moreover, neonatal immune parameters, postnatal clinical outcomes, and long-term infant follow-up data were not available. Therefore, the clinical significance of preserved second-trimester fetal TTR in pregnancies treated with levothyroxine after early TSH elevation could not be directly evaluated.

Despite these limitations, the use of a standardized fetal thymus assessment method, evaluation within a relatively narrow gestational age range, and exclusion of pregnancies with major maternal, fetal, or obstetric complications strengthen the internal consistency of the study. Future prospective studies should incorporate serial maternal thyroid function tests, thyroid autoantibody assessment, levothyroxine dose and adherence data, standardized prospective thymus imaging, and neonatal immune or clinical outcomes.

## Conclusion

In conclusion, second-trimester fetal thymic-thoracic ratio values did not differ significantly between healthy controls and pregnancies receiving levothyroxine treatment after elevated first-trimester TSH levels. These preliminary findings suggest that early-treated maternal TSH elevation may not be associated with measurable fetal thymic size alteration during the second trimester. However, because of the retrospective design, the limited and likely underpowered sample size, and the absence of complete contemporaneous thyroid function, thyroid autoantibody, treatment dose, and adherence data, definitive conclusions regarding the effect of levothyroxine treatment cannot be drawn. Prospective longitudinal studies incorporating serial maternal thyroid function measurements, thyroid autoantibody assessment, standardized fetal thymus imaging, and neonatal immune outcomes are needed to clarify this relationship.

## Data Availability

The research data are described in the article presented.
